# A 50% Reduction of Excitability but Not of Intercellular Coupling Affects Conduction Velocity Restitution and Activation Delay in the Mouse Heart

**DOI:** 10.1371/journal.pone.0020310

**Published:** 2011-06-01

**Authors:** Mèra Stein, Toon A. B. van Veen, Richard N. W. Hauer, Jacques M. T. de Bakker, Harold V. M. van Rijen

**Affiliations:** 1 Division of Heart & Lungs, Department of Medical Physiology, University Medical Center Utrecht, Utrecht, The Netherlands; 2 Division of Heart & Lungs, Department of Cardiology, University Medical Center Utrecht, Utrecht, The Netherlands; 3 Interuniversity Cardiology Institute of the Netherlands, Utrecht, The Netherlands; 4 Heart Failure Research Center, Academic Medical Center, Amsterdam, The Netherlands; University of Frankfurt - University Hospital Frankfurt, Germany

## Abstract

**Introduction:**

Computer simulations suggest that intercellular coupling is more robust than membrane excitability with regard to changes in and safety of conduction. Clinical studies indicate that SCN5A (excitability) and/or Connexin43 (Cx43, intercellular coupling) expression in heart disease is reduced by approximately 50%. In this retrospective study we assessed the effect of reduced membrane excitability or intercellular coupling on conduction in mouse models of reduced excitability or intercellular coupling.

**Methods and Results:**

Epicardial activation mapping of LV and RV was performed on Langendorff-perfused mouse hearts having the following: 1) *Reduced excitability*: *Scn5a* haploinsufficient mice; and 2) *reduced intercellular coupling*: Cx43^CreER(T)/fl^ mice, uninduced (50% Cx43) or induced (10% Cx43) with Tamoxifen. Wild type (WT) littermates were used as control. Conduction velocity (CV) restitution and activation delay were determined longitudinal and transversal to fiber direction during S_1_S_1_ pacing and S_1_S_2_ premature stimulation until the effective refractory period. In both animal models, CV restitution and activation delay in LV were not changed compared to WT. In contrast, CV restitution decreased and activation delay increased in RV during conduction longitudinal but not transverse to fiber direction in *Scn5a* heterozygous animals compared to WT. In contrast, a 50% reduction of intercellular coupling did not affect either CV restitution or activation delay. A decrease of 90% Cx43, however, resulted in decreased CV restitution and increased activation delay in RV, but not LV.

**Conclusion:**

Reducing excitability but not intercellular coupling by 50% affects CV restitution and activation delay in RV, indicating a higher safety factor for intercellular coupling than excitability in RV.

## Introduction

Various factors [1] determine impulse propagation throughout the heart, among which membrane excitability, intercellular coupling, and tissue architecture (i.e. myocyte size [2], collagen [1], and fiber orientation [3]) are most important. An appropriate interplay between these factors is necessary for proper impulse propagation [1], [4]. The effect of modification of these factors on conduction has been extensively investigated [4], [5], [6], [7]. These studies show that if impulse conduction is challenged, either by reducing membrane excitability [5], [7] or intercellular coupling [6], the effect on impulse conduction at basic cycle length is minor. These data suggest that the heart has ‘conduction reserve’ [8], [9], [10], and that single factors determining impulse conduction either need to be modified to the extreme [6], or moderately in combination [4], [5], [7], in order to exceed the myocardial conduction reserve and cause impulse propagation impairment.

Under physiologic conditions, no differences in conduction characteristics exist between the right (RV) and left ventricle (LV). However, when determinants of impulse conduction are comparably altered in RV and LV, impulse impairment occurs preferentially in RV [4], [5], [6], [7]. This suggests that RV conduction reserve is lower than that of LV, leaving the RV more vulnerable to impulse propagation impairment in the mouse heart.

Conduction velocity restitution and activation delay are considered to be important determinants for electrical stability. Abnormal conduction velocity restitution favors the occurrence of ventricular fibrillation as demonstrated by Saumarez et al [11]. Conduction velocity restitution is defined as conduction velocity in dependence of the diastolic interval [12]. At short diastolic intervals less sodium current is available and membrane excitability is reduced. Reduced excitability lowers the safety factor for conduction. Thus, conduction velocity restitution is a measure for the safety of conduction.

Activation delay (between a stimulus and recording site) differs from conduction velocity restitution in that it involves next to activation delay imposed by conduction velocity, a stimulus-to-activation delay. The latter occurs because of: 1. current to load mismatch due to the fact that the initial wave front is small and has to excite a large area of surrounding myocardium and 2. charging cell membranes and activating sodium conductance require time. Due to structural remodeling, current to load mismatch sites frequently occur in diseased hearts and may affect the stimulus to activation delay.

Several studies on hearts from patients with heart disease have shown that SCN5A and Cx43 expression is reduced by approximately 50% [13], [14], [15], [16]. Computer simulations of ionic mechanisms of propagation in cardiac tissue carried out by Shaw and Rudy suggest that a 50% reduction in membrane excitability has more effect on conduction and safety of conduction compared to a 50% reduction in intercellular coupling [17].

For the current study we have performed a retrospective analysis of data of 2 previous studies on mice with a 50% reduction in *Scn5a* expression [5] and on mice with Cx43 expression levels of 50% or 10% [6]. We aimed to find evidence for the preferential role of intercellular coupling compared to excitability to maintain normal conduction and safety of conduction in whole hearts with impaired excitability or intercellular coupling by detailed analysis of conduction delay and conduction velocity restitution in these models.

The study demonstrates that reduced membrane excitability increases longitudinal activation delay and impairs conduction velocity restitution significantly in RV. In LV a similar trend was observed. Reduced intercellular coupling by 50% had neither effect on conduction velocity restitution nor on activation delay in either RV or LV. However, reduction of intercellular coupling to 10% did result in increased activation delay and impairment of conduction velocity restitution in RV. This implies that in the intact mouse heart a 50% reduction of excitability is sufficient to increase activation delay and decrease safety of conduction, but that these effects are only observed after reduction to only 10% intercellular coupling These data are compatible with the results of Shaw and Rudy obtained from a computer model of cardiac propagation [17]. In addition, our data show that the effect of decreased excitability on conduction is more outspoken in RV than LV.

## Materials and Methods

### Animals

Electrophysiologic recordings were analyzed from 2 studies [5], [6].

Study 1, reduced cell-to-cell coupling [6] in young heterozygous Connexin43 (Cx43) mice: Cx43^Cre-ER(T)/flox^ plus carrier (n = 8) in which Cx43 is reduced to 50%. Cx43 expression was further decreased to only 10% by intraperitoneal injections on 5 consecutive days with 4-hydroxytamoxifen. Cx43 expression was unaffected in Cx43^flox/flox^ plus carrier littermates (n = 8), which served as control. Mice were of mixed genetic background of 129P2/OlaHsd-C57BL/6. Cx43 levels were verified by western blotting [6].

Study 2, reduced excitability [5] in young heterozygous *Scn5a* (HZ) mice of C57BL/6 background (n = 8). These animals have 50% reduction of Nav1.5 expression as established by western blotting , corresponding to 50% reduction of the sodium current [18]. Young wild-type (WT) C57BL/6 mice (n = 10) served as control.

The investigation conformed to the guiding principals of the Declaration of Helsinki. All animal experiments were performed after approval by the Utrecht University Animal Ethics Review Committee (approval number 102296).

### Preparation of the hearts

For both studies, mice were anaesthetized by intraperitoneal injection of urethane (2 g/kg bodyweight). The chest was opened and the heart was excised and submerged in Tyrode's solution 17 at 4°C. With the help of a binocular microscope the heart was dissected from the lungs as well as other tissue and the aorta was cannulated. Subsequently, the heart was connected to a Langendorff perfusion setup and perfused at 37°C and perfusion pressure of 80 cm H_2_0. Perfusion buffer composition (in mM): NaCl 90, KCl 3.6, KH_2_PO_4_ 0.92, MgSO_4_ 0.92, NaHCO_3_ 19.2, CaCl_2_ 1.8, glucose 22, creatin 6, taurin 6, insulin 0.1 µM, gassed with 95% O_2_ and 5% CO_2_. In all experiments the heart started to beat immediately after initiating perfusion. Flow rate was approximately 2 ml/min. To ensure proper temperature of the preparation, the heart was placed against a heated (37°C) and continuously moisturized support.

#### Recording of Electrograms during Langendorff Perfusion

**Figure 1:**
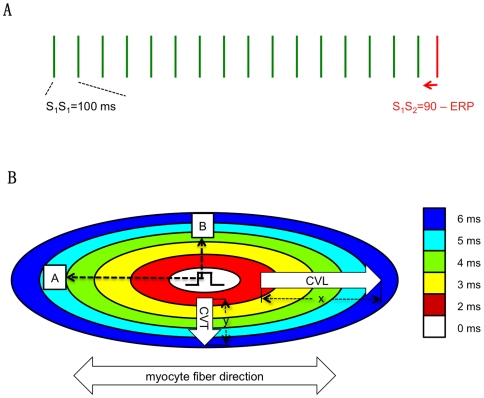
Determination of Conduction Velocity and Activation delay. **A.** The ventricles were stimulated from the center of the grid at S_1_S_1_ stimulation of 100 ms. The stimulation protocol was composed of sixteen basic stimuli followed by 1 premature stimulus (S_1_S_2_). The premature stimulus started at 90 ms and at the subsequent trains, the coupling interval of the premature stimulus was reduced in steps of 5 ms, until the effective refractory period (ERP) was reached, which was defined as the longest possible coupling interval of the premature stimulus that fails to activate the entire heart. **B.** CV parallel (longitudinal; CV_L_) and perpendicular (transverse; CV_T_) to myocyte fiber direction was determined from each activation map. For CVL, the distance between 4 consecutive electrodes parallel to fiber orientation and perpendicular to the isochrones was measured (x) and divided by the time difference (6−2 = 4 ms). Similarly, CVT was determined as Δy/Δt. Activation delay is defined as the local activation time (stimulus is time zero) at a fixed distance from the center of activation origin (stimulus site). Activation delay is registered at two sites (A and B), located on a line parallel to longitudinal and transversal conduction propagation during S_1_S_1_. Subsequently, activation delay is measured at the same sites during the premature stimuli S_1_S_2_. Finally, activation delay is normalized by substracting the activation delays of S_1_S_1_ from activation delay at S_1_S_2_.

Electrical recordings of RV and LV were made with a 247 points unipolar electrode (19×13 grid, spacing 300 µm) as described previously [5], [6]. The ventricles were stimulated from the center of the grid at S_1_S_1_ cycle length of 100 ms. Electrograms were acquired using a 256-channel data acquisition system (Biosemi, Amsterdam). The premature stimulation method (sixteenth basic stimulus followed by 1 premature stimulus) was applied until the effective refractory period was reached; starting at 90 ms, the coupling interval of the premature stimulus S_1_S_2_ was reduced in steps of 5 ms, until the effective refractory period (figure 1A) [6].

#### Data Analysis

The moment of maximal negative dV/dt in the unipolar electrograms was selected as the time of local activation and determined with custom written software based on Matlab (The Mathworks Inc.) [19]. Activation times were used to construct activation maps. Activation maps were constructed during S_1_S_1_ pacing and after each premature stimulus. Of each activation map the following parameters were determined (figure 1B): 1) Conduction velocity parallel (longitudinal; CV_L_) and perpendicular (transverse; CV_T_) to fiber direction. Fiber direction was determined by conventional histology and was perpendicular for RV and oblique for LV with regard to the long axis of the heart (data not shown). Activation times of at least 4 consecutive electrode terminals along lines perpendicular to intersecting isochronal lines (1 ms) were used to calculate conduction velocity.

2) Activation delay is defined as the local activation time (stimulus is time zero) at a distance L from the center of activation origin (stimulus site). Activation delay is registered at two sites (A and B), located on a line parallel to longitudinal and transversal conduction propagation during S_1_S_1_. Activation delay is determined during S_1_S_1_ and for successive premature beats. From these values, absolute activation delays were calculated as follows; Local activation time at site A and B during S_1_S_1_ was subtracted from measured values at S_1_S_2_, defining S_1_S_1_ activation time as ‘zero’, allowing comparison of changes in delay between the groups.

It is important to realize, that even though the absolute increase in delay is measured, this parameter is influenced by two factors: 1) the stimulus-to-activation delay (which may change with prematurity of the stimulus), and 2) activation delay caused by conduction. As such, to measure delay that is independent of the stimulus delay, the conduction velocity was measured as well.

3) Conduction velocity restitution; restitution is the property that, as the diastolic interval of a premature beat varies, the conduction velocity of that beat also varies, typically decreasing with decreasing diastolic interval [20] and is rate dependent. The conduction velocity restitution curve can be measured by plotting conduction velocity along S_1_S_1_ and the S_1_S_2_ coupling interval.

Curves of both ‘conduction velocity restitution’ and ‘activation delay’ were constructed from average values of at least 2 measurements. For specific number of measurements per S_1_S_1_ and S_1_S_2_ coupling intervals, see Tables 1 and 2.

**Table 1:** Reduced membrane excitability group conduction velocity and stimulus-to-activation delay measurements.

	S_1_S_2_	WT RV	HZ RV	WT LV	HZ LV
CV_L_	100	34.9±2.2 (10)	25.8±2.0 (8)§	33.5±2.5 (7)	25.3±2.2 (5)
	90	34.2±2.1 (10)	24.3±1.6 (8)§	32.9±2.4 (7)	22.6±4.1 (5)§
	85	31.9±2.4 (10)	25.4±1.7 (7)	29.1±1.4 (7)	24.3±2.6 (3)
	80	29.9±2.4 (10)	21.9±1.8 (7)§	27.1±1.1 (7)	21.3±1.2 (3)
	75	29.6±2.4 (10)	22.6±1.8 (5)	24.6±3.3 (4)	18.3±1.5 (3)
	70	29.4±1.9 (10)	18.4±2.9 (3)	26.1±2.2 (4)	
	65	29.8±1.7 (8)	27.9±8.2 (2)	26.1±3.9 (3)	
	60	29.3±1.6 (7)	27.1±3.0 (2)		
	55	28.3±3.7 (4)			
CV_T_	100	21.8±2.2 (10)	17.9±1.4 (8)	19.6±0.9 (7)	15.2±1.6 (5)
	90	22.4±2.9 (10)	17.1±0.9 (8)	18.6±0.9 (7)	15.4±2.0 (5)
	85	20.8±2.1 (10)	15.5±1.1 (7)	18.2±0.9 (7)	15.2±1.9 (3)
	80	19.5±2.3 (10)	15.2±1.3 (7)	17.6±1.1 (7)	17.2±2.0 (3)
	75	19.8±1.7 (10)	14.5±1.0 (5)	15.1±1.7 (4)	18.2±1.0 (3)
	70	20.2±2.1 (10)	17.6±0.5 (3)	14.1±2.1 (4)	
	65	21.2±2.2 (9)	18.6±3.2 (2)	12.6±2.3 (3)	
	60	20.8±3.1 (7)	20.5±3.5 (2)		
	55	24.1±3.5 (4)			
StAD_L_	100	0.0±0.0 (10)	0.0±0.0 (8)	0.0±0.0 (7)	0.0±0.0 (5)
	90	0.6±0.2 (10)	2.3±1.0 (8)	0.6±0.2 (7)	2.1±0.7 (5)
	85	1.0±0.2 (10)	2.6±0.6 (7)§	1.5±0.4 (7)	2.7±0.7 (3)
	80	1.7±0.3 (10)	3.7±0.8 (7)§	2.4±0.5 (7)	4.2±0.9 (3)
	75	2.3±0.4 (10)*	5.9±2.4 (5)	3.3±0.32(4)*	6.3±0.9 (3)
	70	4.1±0.6 (10)	5.8±0.2 (3)§	6.1±0.4 (4)*	
	65	5.4±1.0 (9)	8.5±2.0 (2)	10.2±2.6 (3)	
	60	5.7±0.6 (7)	14.0±2.5§		
	55	7.8±1.7 (4)			
StAD_T_	100	0.0±0.0 (10)	0.0±0.0 (8)	0.0±0.0 (7)	0.0±0.0 (5)
	90	0.7±0.3 (10)	2.1±1.2 (8)	0.7±0.3 (7)	2.1±0.5 (5)
	85	1.2±0.3 (10)	2.4±0.6 (7)	2.4±0.8 (7)	3.2±0.6 (3)
	80	2.5±0.6 (10)	3.6±0.7 (7)	3.4±1.0 (7)	6.2±1.9 (3)
	75	2.6±0.7 (10)	6.7±2.8 (5)	4.0±1.4 (4)	8.2±2.2 (3)
	70	4.7±1.1 (10)	8.0±2.1 (3)	5.8±1.1 (4)	
	65	5.3±1.3 (9)	11.8±0.8 (2)	7.3±1.7 (3)	
	60	5.8±0.9 (7)	16.8±0.3 (2)§		
	55	6.6±0.9 (4)			

All values are mean±SEM. WT – wild-type, HZ – heterozygous. S_1_S_2_ coupling interval is in ms. CV_L_/_T_ – longitudinal/transverse conduction velocity (cm/s); StAD_L_/_T_ – stimulus-to-activation delay longitudinal/transverse (ms).

* intra-variable differences: P<0.05 between consecutive S_1_S_2_ and S_1_S_2_-5 ms.

Inter-variable differences are within either RV or LV:

§ P<0.05 between wild-type and heterozygous animals.

**Table 2:** Reduced intercellular coupling group conduction velocity and stimulus-to-activation delay measurements.

	S_1_S_2_	100% Cx43 RV	50% Cx43 RV	10% Cx43 RV	100% Cx43 LV	50% Cx43 LV	10% Cx43 LV
CV_L_	100	31.0±1.6 (16)	33.0±2.2 (8)	24.8±3.3 (9)	32.8±3.3 (9)	37.5±3.3 (7)	31.9±2.3 (6)
	90	29.2±1.6 (16)	31.3±1.5 (8)	22.6±2.7 (9)¥,†	31.5±2.9 (9)	33.0±2.9 (7)	26.8±2.8 (6)
	85	28.1±1.4 (16)	31.5±1.7 (8)	21.5±2.8 (9)¥,†	30.0±3.7 (9)	31.4±3.0 (7)	24.2±3.0 (6)
	80	28.0±1.5 (16)	30.4±1.5 (8)	18.2±2.7 (8)¥,†	24.8±2.3 (9)	30.8±3.3 (7)	22.2±3.2 (6)
	75	28.5±1.4 (15)	28.5±1.7 (8)	15.6±3.0 (8)¥,†	22.8±1.8 (6)	29.3±3.7 (6)	20.5±2.7 (6)
	70	25.9±1.3 (12)	28.1±1.8 (7)	15.9±2.4 (9)¥,†	22.1±4.2 (5)	26.7±2.8 (5)	23.0±2.0 (4)
	65	24.9±1.1 (12)	27.1±1.4 (3)	14.8±2.9 (7)¥,†	20.0±3.8 (4)	23.3±2.2 (4)	19.5±1.6 (4)
	60	24.0±1.2 (8)		14.7±4.1 (4)¥		20.3±2.9 (4)	
	55	20.6±2.2 (4)		15.9±4.2 (3)			
CV_T_	100	24.6±1.1 (16)	21.5±1.9 (8)	14.3±2.0 (9)¥,†	18.2±1.2 (9)	18.5±1.6 (7)	13.3±1.5 (6)
	90	24.0±1.2 (16)	21.2±1.62 (8)	12.7±1.5 (9)¥,†	17.5±1.4 (9)	18.7±1.4 (7)	13.5±1.5 (6)
	85	23.7±1.1 (16)	20.2±1.63 (8)	12.5±2.0 (9)¥,†	16.2±1.0 (9)	17.4±1.6 (7)	14.4±1.5 (6)
	80	23.6±1.2 (16)	**1.6 (8)**	12.7±2.3 (8)¥	15.4±0.8 (9)	17.5±1.2 (7)	12.3±1.9 (6)†
	75	21.8±1.1 (15)	19.4±1.7 (8)	11.7±1.7 (8)¥,†	14.7±0.8 (6)	16.0±1.5 (6)	13.6±1.4 (6)
	70	21.9±1.1 (12)	19.5±1.7 (7)	10.6±1.8 (9)¥,†	16.2±1.7 (5)	13.0±1.6 (5)	12.2±1.7 (4)
	65	21.0±1.4 (12)	19.1±2.9 (3)	11.5±2.4 (7)¥	13.2±1.3 (4)	13.5±2.2 (4)	11.5±1.2 (4)
	60	17.7±0.7 (8)		10.2±2.2 (4)¥		14.5±1.6 (4)	
	55	16.8±0.4 (4)		11.8±3.1 (3)			
StAD_L_	100	0.0±0.0 (16)	0.0±0.0 (8)	0.0±0.0 (9)	0.0±0.0 (9)	0.0±0.0 (7)	0.0±0.0 (6)
	90	0.4±0.2 (16)	0.9±0.3 (8)	0.4±0.1 (9)	1.1±0.4 (9)	0.3±0.2 (7)	0.8±0.3 (6)
	85	1.1±0.5 (16)	1.4±0.6 (8)	1.1±0.3 (9)	2.1±0.6 (9)	0.3±0.2 (7)§	1.6±0.4 (6)
	80	1.6±0.7 (16)	1.7±0.7 (8)*	1.6±0.5 (8)	2.4±0.4 (9)	0.9±0.2 (7)	2.8±1.0 (6)
	75	2.0±0.4 (15)	2.8±0.6 (8)	3.1±0.9 (8)	4.3±1.1 (6)	1.5±0.2 (6)	3.1±0.5 (6)
	70	2.8±0.5 (12)*	4.7±0.8 (7)	4.4±1.6 (9)	4.7±0.9 (5)	3.3±0.7 (5)	5.6±1.5 (4)
	65	5.0±0.9 (12)	6.2±2.0 (3)	7.6±2.7 (7)	4.6±0.8 (4)	4.1±0.6 (4)	8.8±2.3 (4)
	60	5.9±1.0 (8)		9.4±3.1 (4)*		10.1±1.7 (4)	
	55	7.3±0.9 (4)		20.8±12.7 (3)			
StAD_T_	100	0±0.0 (16)	0.0±0.0 (8)	0.0±0.0 (9)	0.0±0.0 (9)	0.0±0.0 (7)	0.0±0.0 (6)
	90	0.6±0.2 (16)	0.8±0.3 (8)	0.6±0.2 (9)	1.4±0.7 (9)	0.6±0.3 (7)	1.2±0.5 (6)
	85	0.9±0.3 (16)	1.4±0.4 (8)	1.7±0.3 (9)	2.9±0.9 (9)	0.7±0.2 (7)	1.9±0.3 (6)
	80	1.5±0.5 (16)	1.9±0.5 (8)	2.8±0.8 (8)	3.5±0.9 (9)	1.9±0.7 (7)	3.0±0.7 (6)
	75	2.0±0.5 (15)	2.8±0.6 (8)	4.5±1.0 (8)¥	4.8±1.0 (6)	2.4±0.5 (6)*	4.4±1.0 (5)
	70	2.9±0.4 (12)*	4.6±1.0 (7)	5.8±1.0 (9)¥	6.0±1.3 (5)	4.1±0.6 (5)	5.8±1.3 (4)
	65	4.6±0.7 (12)	5.5±1.3 (3)	10.6±2.3 (7)¥	7.5±1.1 (4)	7.0±1.8 (4)*	9.0±2.2 (4)
	60	6.0±0.8 (8)		11.3±3.5 (4)		12.1±1.7 (4)	
	55	7.1±0.7 (4)		19.5±11.2 (3)			

All values are mean±SEM. S1S2 coupling interval is in ms. CVL/T – longitudinal/transverse conduction velocity (cm/s); StADL/T – stimulus-to-activation delay longitudinal/transverse (ms).

*intra-variable differences: P<0.05 between consecutive S1S2 and S1S2-5 ms.

Inter-variable differences are within either RV or LV:

§ P<0.05 between 100% and 50% Cx43.

† P<0.05 between ventricles with 50% and 10% Cx43.

¥ P<0.05 between ventricles with 100% and 10% Cx43.

To facilitate comparison between activation maps, similar colors in all figures represent equal activation time.

### Statistics

CV restitution and activation delay curves data were analyzed using two-way repeated measurements ANOVA with a LSD post-hoc test (corrected for comparisons made).

Probability values of *P*≤0.05 were considered statistically significant. Data was analyzed using SPSS 13.0 software.

## Results

### Conduction Velocity Restitution and Activation Delay

Tables 1 and 2 provide mean±SEM values of the study groups during S_1_S_1_ and S_1_S_2_ coupling intervals. Table 3 supplies a summary of RV and LV conduction velocity restitution and activation delay of the groups.

**Table 3:** Summary of right and left ventricular CV restitution and activation delay.

Right Ventricle	Longitudinal Conduction				Transversal Conduction	
	CV restitution		Activation Delay		CV restitution	Activation Delay
Reduced Excitability (50%)	k	lPrior to block	k	lPrior to block	∼	∼
Reduced Coupling (50%)	∼		∼		∼	∼
Reduced Coupling (10%)	l		∼		l	l
**Left Ventricle**						
Reduced Excitability (50%)	∼		∼		∼	∼
Reduced Coupling (50%)	∼		∼		∼	∼
Reduced Coupling (10%)	∼		∼		∼	∼

k = increased; l = decreased; ∼ = unchanged.

### Reduced Membrane Excitability

**Figure 2:**
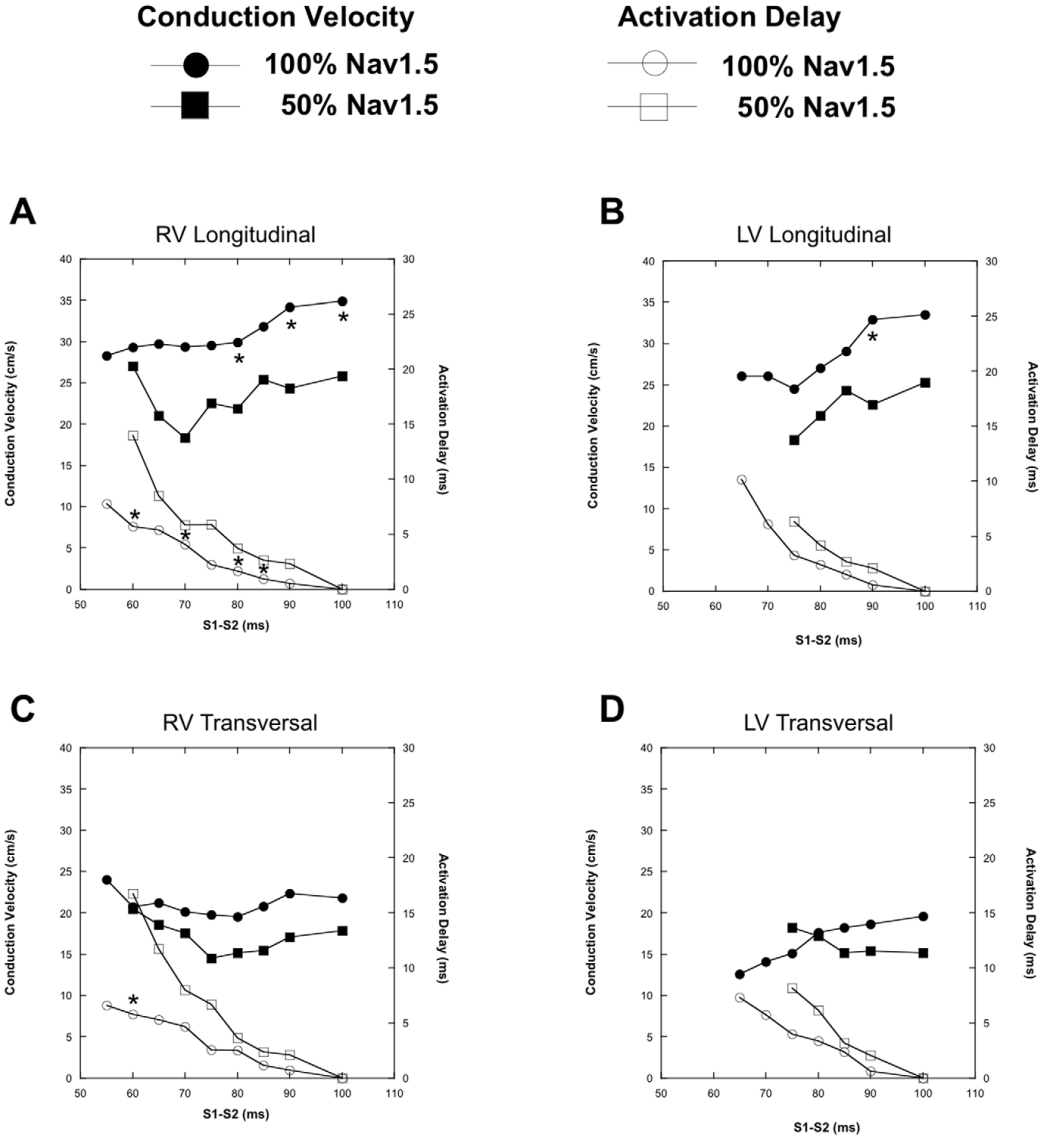
CV restitution and activation delay curves of mice demonstrating reduced membrane excitability. CV restitution (solid markers) and activation delay (open markers) curves of WT (circles) and *Scn5a* HZ (squares). Hearts are paced at S_1_S_1_ of 100 ms with S_1_S_2_ coupling interval reduced in steps of 5 ms until the effective refractory period. A and B demonstrate the RV and LV CV_L_ restitution and activation delay curves, C and D show the curves during transverse propagation.

Conduction velocity restitution (closed markers) and activation delay (open markers) curves of WT (circles) and *Scn5a* HZ (squares) animals are depicted in figure 2 (A to D). In RV, CV_L_ restitution was significantly affected by the reduced membrane excitability (solid markers in figure 2A). Decreased CV_L_ in RV was mainly seen during long S_1_S_2_ coupling intervals. Interestingly, with S_1_S_2_ coupling intervals near the refractory period, CV_L_ increased in the *Scn5a* HZ mice rather than further decreased as occurred in WT animals. This sudden increase in CV_L_ in RV of *Scn5a* HZ animals close to the effective refractory period was accompanied by a significant increase in activation delay (open squares in figure 2A). The increased CV_L_ restitution at S1-S2 between 70 and 60 ms, was accompanied by a steeper course of longitudinal activation delay. CV_L_ restitution and longitudinal activation delay of the *Scn5a* HZ were not significantly affected in LV as compared to WT (figure 2B, solid and open markers, respectively).

Interestingly, transverse impulse propagation was not significantly affected in both RV and LV by reduced *Scn5a* expression (figure 2C and 2D, respectively), although the trend was similar to changes in RV. Comparable to CV_L_, CV_T_ in RV increased at short S_1_S_2_ coupling intervals, which was accompanied by a steeper increase in the transverse activation delay of RV. CV_T_ restitution and transverse activation delay in LV of the HZ animals were not significantly affected compared to WT animals.

**Figure 3:**
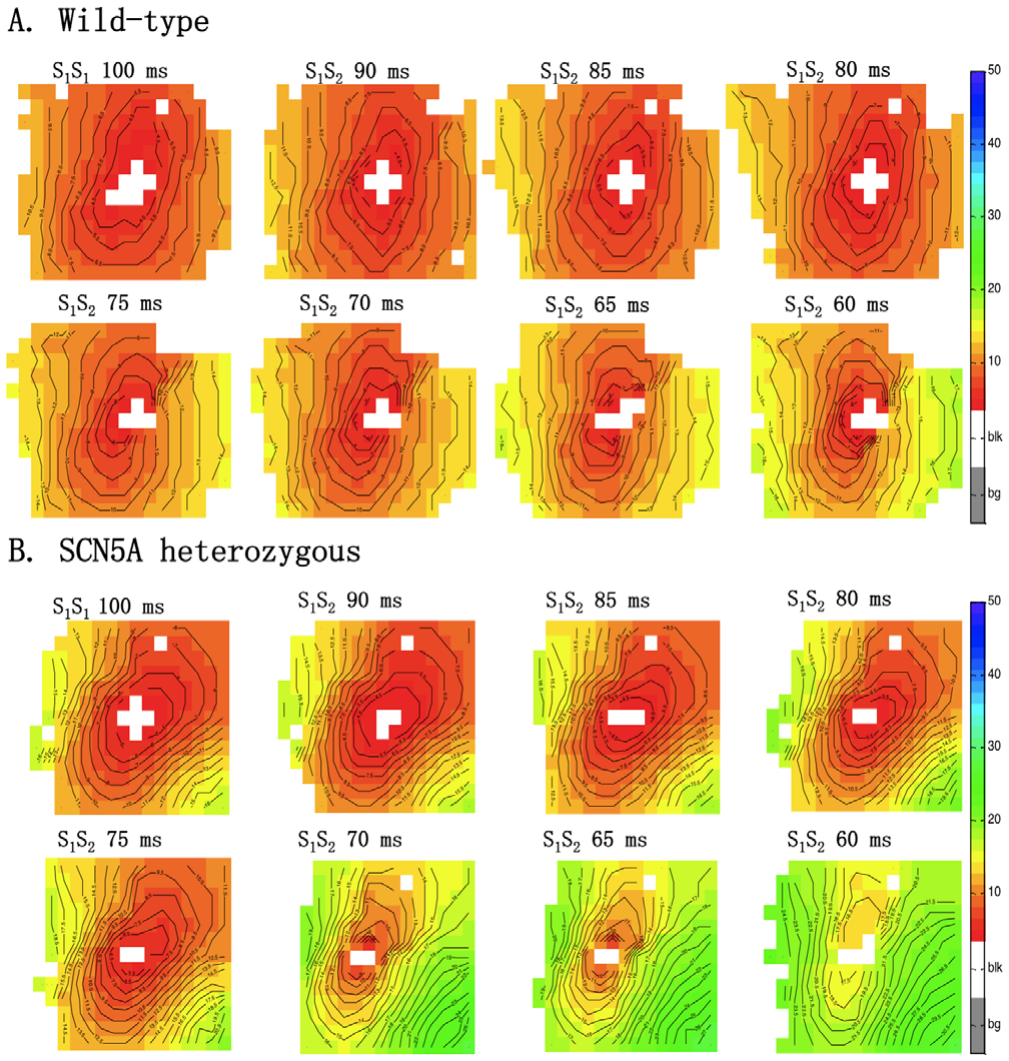
Activation maps of wild-type and *Scn5a* heterozygous RV. RV activation maps of WT (panel A) and *Scn5a* heterozygous (panel B) mice paced at S_1_S_1_ of 100 ms and during S_1_S_2_ activation with 5 ms decrement until the effective refractory period is reached. Isochronal lines are set to 1 ms. Red denotes earliest activation, blue latest. Equal color represents equal activation times.

Figure 3 shows activation maps of a WT (panel A) and *Scn5a* HZ (panel B) RV for different coupling intervals of the premature stimulus. In WT animals the delay between the stimulus and earliest activation hardly changed with shortening of the coupling interval (earliest activation started within 5 ms after the stimulus). However, in the *Scn5a* HZ mice activation delay was significantly greater. For example, earliest activation at an S_1_S_2_ interval of 60 ms starts 15 ms after the stimulation was applied.

### Reduced Intercellular Coupling

**Figure 4:**
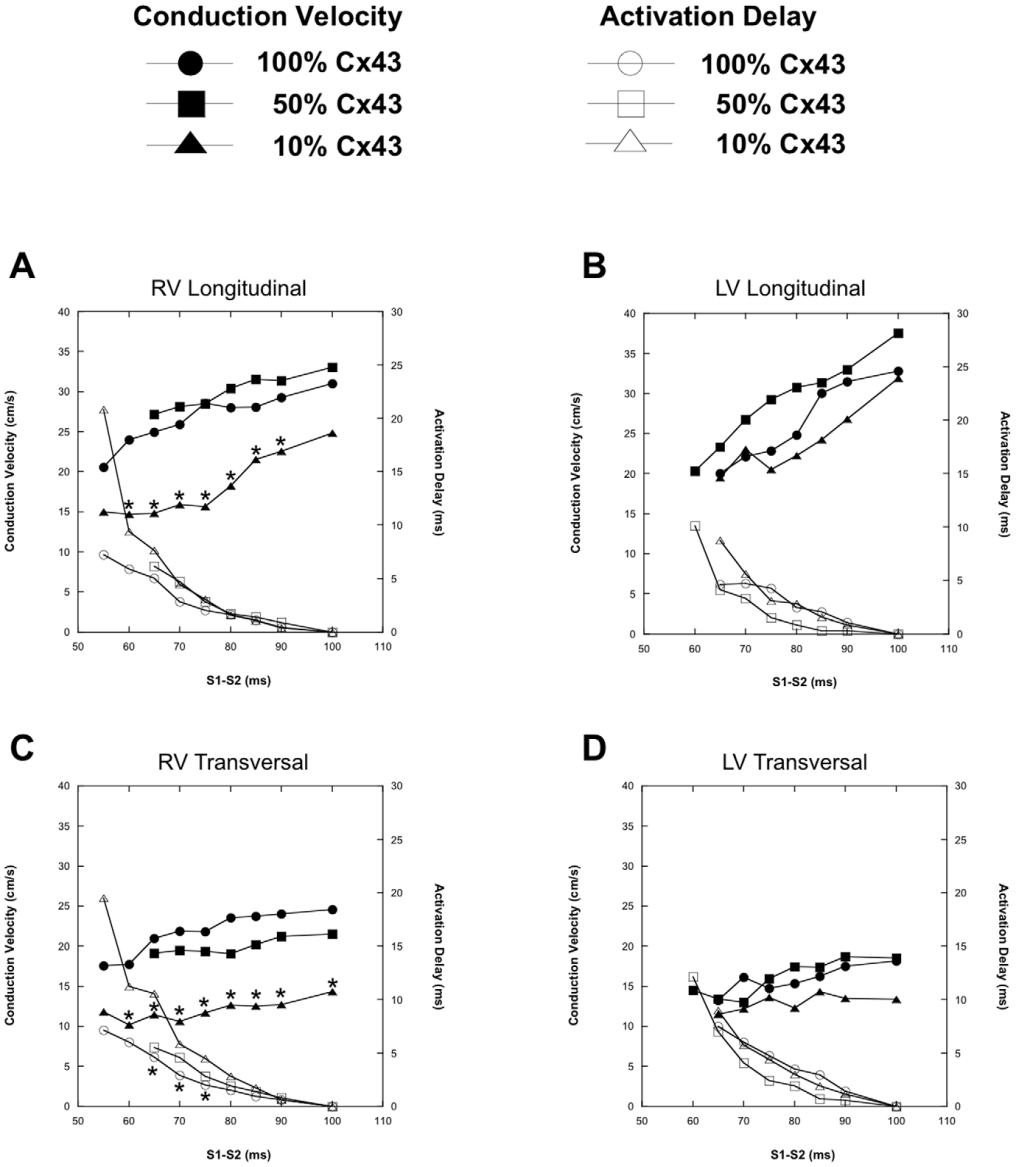
CV restitution and activation delay curves of mice with reduced intercellular coupling. CV restitution (solid markers) and activation delay (open markers) curves of Cx43^flox/flox^ animals (control levels of Cx43, circles), and animals with reduced intercellular coupling of 50% (squares) and 10% (triangles). Hearts are paced at S_1_S_1_ of 100 ms with S_1_S_2_ coupling interval reduced in steps of 5 ms until the effective refractory period. A & B are RV and LV longitudinal CV restitution and activation delay curves. C and D demonstrate the transverse RV and LV CV restitution and activation delay curves.

Figure 4 shows conduction velocity restitution (solid markers) and activation delay (open markers) curves of Cx43^flox/flox^ animals (control levels of Cx43, circles), animals with 50% reduced intercellular coupling by (squares), and animals with only 10% Cx43 expression (triangles). A 50% reduction in Cx43 expression did alter neither conduction velocity restitution nor activation delay of both longitudinal and transverse propagation, of both RV and LV, as compared to animals with 100% Cx43. However, reduction of Cx43 to 10% in RV resulted in significantly reduced conduction velocity restitution, both longitudinally and transversely. Activation delay was increased in both directions in RV, but only significantly in transverse direction (Figure 4A&C). In LV of hearts expressing only 10% Cx43, conduction velocity restitution and activation delay were not different from 50% or 100% Cx43 (Figure 4B&D).

**Figure 5:**
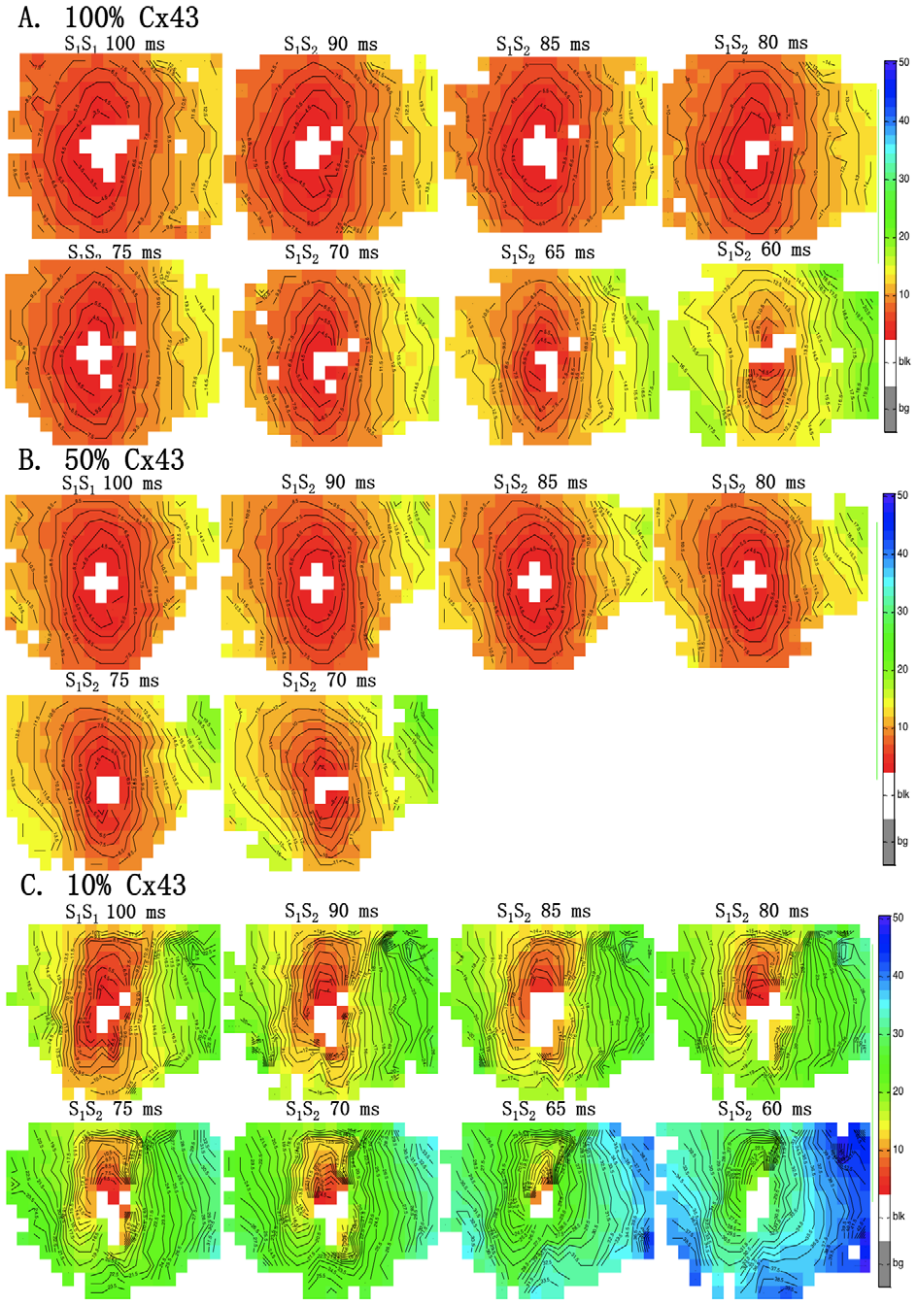
Activation maps of 100% , 50% and 10% Cx43 for RV. The figure shows RV activation maps for 100% Cx43 (A), 50% Cx43 (B), and 10% Cx43 (C). Either S_1_S_1_ of 100 ms or S_1_S_2_ coupling interval (decrement of 5 ms) is specified above the activation map. Isochronal lines are at 1 ms intervals. Red denotes earliest activation, blue latest. Equal color represents similar activation time.

Figure 5 demonstrates activation patterns of RV with 100% (panel A), 50% (panel B), and 10% Cx43 expression (panel C). An increase in total activation delay in the recording area was virtually absent till a coupling interval of 70 ms in animals with 100%, 50%, and 10% Cx43 expression.

## Discussion

Conduction velocity restitution and activation delay were examined in mice with reduced excitability, and reduced intercellular coupling. The main findings of this study are: 1) During longitudinal propagation conduction velocity restitution decreases and activation delay increases if excitability is reduced by 50%, but only significantly in RV. Similar trends were observed during transverse propagation in RV and propagation in LV; 2) reduced intercellular coupling by 50% did not affect conduction velocity restitution nor activation delay, but reduction to 10% resulted in a significant decrease in conduction velocity restitution during both longitudinal and transversal propagation in RV. Activation delay at 10% Cx43 was increased in RV, but only significant for transverse propagation. In LV, conduction velocity restitution and activation delay was similar for 100%, 50% and 10% Cx43 levels.

These results, which show that cell-to-cell coupling is more robust with regard to conduction than excitability, are compatible with the observations made by Shaw and Rudy in a computer model of propagation. In addition, our animal experiments show that RV is more vulnerable for changes in excitability than LV.

### Reduced Membrane Excitability

Previous studies have shown that the effect of reduced membrane excitability [5] or reduced intercellular coupling [6] on mouse heart electrophysiology are different. However, one common denominator exists between these mouse models: RV impulse propagation impairment is significantly affected, in contrast to LV [4], [5], [6], [21]. Activation delay and conduction velocity restitution of the genetically modified mice demonstrate a higher vulnerability of RV for electrophysiological changes.

Reduced excitability has a peculiar effect on conduction velocity restitution and activation delay in RV. At S1S1 and long S_1_S_2_ coupling intervals CV_L_ is decreased in RV of HZ mice, while CV_T_ is not significantly altered. This fits well to the theory of Spach of discontinuous conduction in the heart [22]. Conduction in the long and low resistive axis of the myocytes was more vulnerable to premature stimulation than in transverse directions and conduction block preferentially occurred in the long axis. This is explained by source-sink relationships between excited (source) and unexcited (sink) cells in the propagation path (for a review see [1]). The resistance of the longitudinal conduction path is low, while that of the transverse pathway is high, due to the shape of the myocytes and preferential location of gap junctions at the intercalated disk [23]. As a result, more current is needed in the longitudinal direction for activation than transverse, resulting in higher vulnerability to longitudinal conduction slowing during reduced sodium current due to premature stimulation, and in our study, combined with genetic reduction of sodium channel expression.

When the coupling interval is further reduced (Fig. 2A), activation delay increases, while CV_L_ of HZ in RV is no longer significantly decreased compared to WT RV. Shaw and Rudy discussed extensively the mechanism of reduced membrane excitability on conduction velocity [17]. It is evident that when membrane excitability is reduced, conduction velocity will decrease. However, at shorter S_1_S_2_ coupling intervals activation delay increases (up to 25 ms delay prior to conduction block), while conduction velocity does not further decrease. It is plausible that during such long delay additional support to conduction is delivered from another inward current, i.e. the L-type Calcium current (I_Ca(L)_) [17]. In well-coupled fibers excitability and conduction are determined by I_Na_. However, under conditions of reduced membrane excitability, the fast nature of sodium channel inactivation allows contribution of I_Ca(L)_, the increase in axial current delivered to depolarize downstream cells [17]. Another explanation may result from the high activation delay, which allows greater recovery time from inactivation for the Na*_v_*1.5 channels in the myocytes distal of the stimulation site, resulting in even higher conduction velocity at shorter coupling intervals.

### Reduced Intercellular Coupling

Reduction of intercellular coupling by 50% has little effect on conduction velocity restitution and activation delay. Although impulse delay in RV increased and conduction velocity restitution decreased at 50% Cx43 compared to wild type, these alterations were not significant. These changes became significant after reduction to 10%. Both longitudinal and transverse conduction velocity restitution were decreased in RV and activation delay increased, albeit only significant for transverse propagation. Jongsma and Wilders [24] demonstrated in a computer model of impulse propagation that moderate reduction of gap junctional coupling has little or no effect and that large reductions of intercellular coupling are required for significant reduction of conduction velocity. Also in the study of Shaw and Rudy the reduction of conduction velocity at a 50% reduction of intercellular coupling is small (approximately 15%), but reduction of intercellular coupling to 10% leads to a ∼60% decrease in conduction velocity. These data are compatible with experimental findings in Cx43 knockout mice, which show that a 50% reduction in Cx43 expression does not affect conduction, but is significantly reduced by 19 (longitudinal) and 41% (transversal) after reduction to 10% Cx43 expression [6], [25]. More importantly, changes in gap junctional conductance (g_j_) have a greater impact on transverse conduction. CV_L_ is rather insensitive to changes in effective g_j_ [24]. Spach *et al* [2] demonstrated that the mean activation delay between cells during transverse propagation is significantly higher than during longitudinal propagation. Due to the nature of gap junction distribution in the adult myocardium, in the longitudinal direction tight end-to-end coupling between the myocytes ensures minor cell-to-cell delay during longitudinal propagation. While during transverse propagation, more cell-to-cell borders are present and the increased lateral detachment produced a prominent increase in mean lateral cell-to-cell delay [2]. These studies explain the impact of severe uncoupling on the reduced CV_L_ restitution which is not accompanied by an increased activation delay.

### Limitations

The study shows that conduction velocity restitution has methodological limitations. As we try to investigate the effect of reduced membrane excitability and reduced intercellular coupling on activation delay and conduction velocity restitution, we apply trains of 16 S_1_S_1_ followed by a decremented S_1_S_2_ coupling interval (until the effective refractory period). This method, however, generally applied in measurements of cardiac electrical restitution is expected not to affect activation delay. Our measurements, however, show that under certain conditions such as reduced excitability, activation delay increases with shortening of the coupling interval. Thus, conduction velocity may increase at shorter coupling intervals because the activation delay allows more time for the tissue to recover. Furthermore, slight differences in genetic background between the models may have influenced the results.

The effect of combined reductions in both sodium current and electrical coupling on conduction velocity and arrhythmogenesis was recently studied by our group [26]. Reduction of both electrical coupling and excitability resulted in normal conduction velocity parallel to fibre orientation but in pronounced conduction slowing transverse to fibre orientation in RV only, although this did not affect arrhythmogeneity. Although these data are available for retrospective analyses, they have not been added, because the mouse model used was different. In this study, the reduced peak sodium current was based on the 1798insD mutation [27]. Although peak sodium current is indeed decreased in this model, it is also characterized by increased late sodium current and AP prolongation. The latter may strongly influence the restitution curves.

### Conclusion

The mechanism by which reduced membrane excitability and reduced intercellular impairs impulse conduction is mainly derived from results obtained from computer simulations. In this study we demonstrate experimentally the effect of alterations in impulse conduction determinants in Langendorff-perfused mouse hearts and the different effects of these alterations exerts on impulse propagation impairment.

These experiments on intact myocardium underpin computer simulations which show that reduction of excitability has far greater impact than reduction of intercellular coupling. In addition, the data demonstrate that vulnerability of RV for changes in excitability is greater than of LV.
